# New and novel intrinsic host repressive factors against HIV-1: PAF1 complex, HERC5 and others

**DOI:** 10.1186/1742-4690-9-19

**Published:** 2012-03-09

**Authors:** Mudit Tyagi, Fatah Kashanchi

**Affiliations:** 1 National Center for Biodefense and Infectious Diseases, George Mason University, Discovery Hall, Room 182, University Blvd. MS 1H8, 10900 Manassas, VA, USA

**Keywords:** Host restriction factors, HIV, Interferon, Therapy

## 

Human Immunodeficiency Virus (HIV), a retrovirus, is the etiological agent of acquired immunodeficiency syndrome (AIDS). AIDS is characterized by the failure of the immune system to protect against not only HIV but also common secondary viral and bacterial infections. Viruses such as HIV-1 are intracellular pathogens that require host cell machinery to maintain and generate new progeny. In that process, viruses disturb the normal components of intrinsic immune responses within infected cells. After HIV infection, the expression of various cellular restriction factors is notably up-regulated due to interferon activation, partly because genes for most of these restriction factors such as APOBEC3, TRIM, BST2/Tetherin, contain interferon-responsive promoters. Importantly, these molecules have been focused on for both elite controllers as well as long-term non-progressor AIDS patients.

The best characterized HIV restriction factors are encoded by the APOBEC3 and TRIM gene families. The interplay between these cellular and viral proteins appears to be an important factor in deciding the ultimate disease outcome. Interestingly, HIV has also armed itself to counter various cellular intrinsic defense mechanisms, thereby overcoming the intrinsic responses mounted by the host. Therefore, today there is an intense effort in the HIV/AIDS field to define both important host-encoded antiviral restriction factors and viral counter-defense mechanisms that play key roles in the pathogenesis. Along these lines, the list of both restriction factors (proteins that counter specific viral proteins) and repressive factors (inhibitors of HIV life cycle) has been growing continuously. These factors not only include APOBECs, TRIM5α, p21/waf1, and SAMHD1, which act on the incoming virus [1], but also TRIM22, TRIM28/KAP1, and BST2/Tetherin, which exert their effect on post integration, assembly and budding.

In a recent manuscript by Liu et al., the authors utilized an siRNA screening method to knockdown expression of 19,121 human genes [2]. They specifically looked for knockdown of restriction factors that would rescue the early stages of HIV replication from post entry to integration. The assay utilized viral pseudotypes HIV89.6R and HIV8.2N which are capable of a single round of infection. To optimize the screening, they used negative control siRNAs targeting cyclophilin B, PLK1 and GFP in target HeLa-CD4 cells. They identified 114 genes that affected a wide range of cellular activities that could be classified as genes that defend against retroviral invasion. These factors fall into various functional categories such as receptor signaling, vesicle trafficking, transcription, mRNA processing, DNA/RNA surveillance, cross-nuclear membrane transport and ubiquitination. Interestingly, they did not observe the presence of the classical RNA-recognizing PRRs such as toll like receptors TLR3, 7 or 8 or RIG-1 like receptors or MDA-5 in their screening. Genes such as *AP2M1, DNM2, SETDB1, PAF1, CTR9 and RTF1 *were identified and further characterized. Some of the gene products were previously shown to be involved in the regulation of endocytosis (AP2M1 and DNM2), HIV pre-integration complex transportation from cytoplasm to nucleus (NPIP, an interacting partner of nuclear pore components such as NUP62), PAF 1 complex and protein methylase (SETDB1).

Their detailed investigation demonstrated the role of PAF1 complex as an important repressive factor during intrinsic defense against HIV infection. Specifically, the PAF1 complex appears to block HIV replication during the early events from post entry to integration of proviral DNA into host genome. Intriguingly, they found that PAF1 complex is present ubiquitously in various cell lineages, which are common targets of HIV that include monocytes, macrophages and T lymphocytes. Moreover, PAF1 complex not only effectively represses HIV infection but also inhibits infection of evolutionarily similar retroviruses such as HIV-2 and SIV.

SETDB1, another interesting protein identified, harbors enzymatic activity as lysine methyltransferase. The SETDB1 possesses specificity for lysine 9 of histone H3 and plays an important role in silencing transcription by depositing specific histone marks, namely H3K9me2-3 during cell differentiation [3,4]. Additionally, SETDB1 has been shown to induce Tat methylation, which results in reduced viral production [5], and on the other hand methyl transferase inhibitors (similar to siRNA used here) lead to the enhancement of virus production [6]. The finding of SETDB1 in the screen of Li et al. further establishes the role of TRIM28 during proviral integration, which it primarily executes by recruiting SETDB1 to the pre-integration complex [7]. It will be interesting to determine how this enzyme regulates the activity of other viral pre-integration proteins such as RT, Gag or IN on specific lysine residues, which could acquire both acetylating and methylation marks.

In another article by Woods at al., the authors confirmed the role of HECT domain and RCC1-like domain-containing protein 5 (HERC5), an interferon inducible gene that restricts the early stages of HIV assembly [8]. Cells expressing HERC5 released 4.0-fold less infectious virus than the control cells after a single round of replication. Furthermore using published databases, the authors found that HERC5 expression is significantly increased in patients in acute and chronic stages of infection but not in non-progressors. Collectively, the data are consistent with previous reports [9-11], where HERC5 restriction is different from the well established anti-HIV-1 activities of ISG15-only expression.

**Figure 1:**
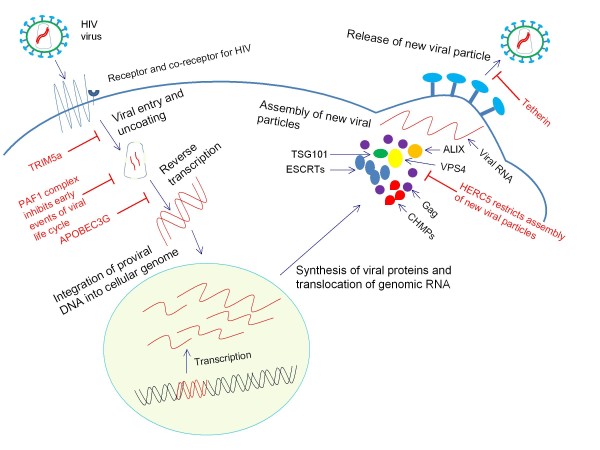
**Two new host repressive factors which inhibit HIV life cycle at different steps**. PAF1 complex seems to inhibit early events of viral life cycle from reverse transcription to integration step. On the other hand HERC5 appears to act at the later part of viral life cycle, that is during the earlier stage of new viral particle assembly, most probably by regulating the ISGylation of Gag protein of HIV.

Thus, these studies add a few more candidates to the ever growing list of HIV repressive and restriction factors that inhibit HIV life cycle at various stages (Figure 1). Taken together, these investigations are novel and open the door for a better understanding of how host cellular factors control infection. Future confirmatory experiments using primary infections of T-cells *vs*. macrophages and field isolate of the virus to elucidate viral targets for each of these important factors and to elicit possible escape mutants to their restriction will shed more light on this fascinating area of research.
